# A new optical pH sensor based on a mixture of Alizarin and Orange dyes immobilized on an agarose membrane

**DOI:** 10.1016/j.mex.2023.102462

**Published:** 2023-10-23

**Authors:** Kamal Alizadeh, Behrooz Rezaei, Hadi Nemati

**Affiliations:** Faculty of Chemistry, Lorestan University, 6813717133, Khorramabad, Iran

**Keywords:** Agarose membrane, pH optical sensor, Alizarin, Orange dye, Epoxy-activated agarose membrane

## Abstract

Two techniques, including chemical immobilization of a combination of two indicators of Alizarin and Orange dyes and the epoxy activation of the agarose membrane, were used for the preparation of a new optic pH sensor. For this purpose, the mentioned dyes were immobilized on agarose support activated by an epoxy, followed by optimizing the impacts of the coupling pH, as well as the ratio and concentration of the two dyes. The sensor was set up in a flow cell and effectively employed for online pH calculations. The new optic pH sensor could be applied between 4.5 and 11pH values. The sensor could quickly respond to pH alterations in nearly 25 s. The sensor's response is adjustable and replicable. Ionic strengths of up to 0.5 mol L^−1^ could have no meaningful impact on the response signal. In addition, no proof of any signal drift or dye leaching was detected over a three-month period.

• The chemical immobilization of two indicators on agarose membranes activated by an epoxy could lead to a sensitive optic pH sensor for a wide range of pH.
• The intended sensor was mounted in a flow cell and effectively utilized for the purpose of online pH measurement.
• The suggested optode was employed to determine pH in real samples of water.

## Graphical abstract

Changes related to the UV–Vis absorbance of two indicators of Alizarin and Orange dyes are detected with increasing pH.

**Figure:**
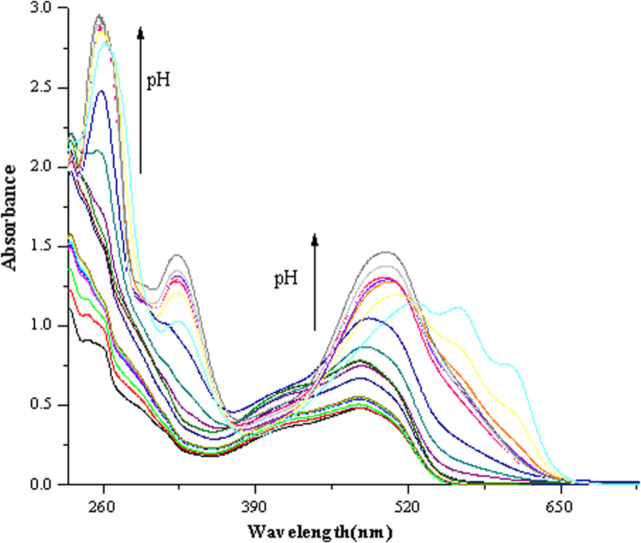

Specifications table

**Table:** 

Subject area	Chemistry
More specific subject area	Two dyes on optically transparent agarose membrane
Name of your method	Epoxy-activated agarose membrane
Name and reference of original method	Wide range pH optical sensor
Resource availability	N/A

## Method details

### Background

**Fig. 1:**
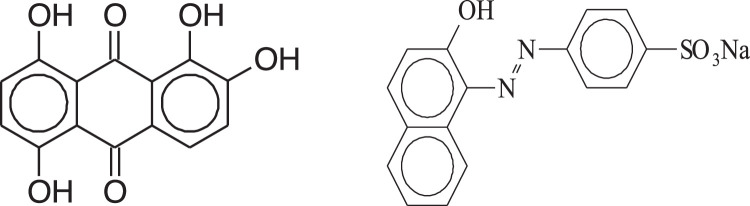
Molecular structures of the applied chemicals: Alizarine (left) and Orange (right).

Optical pH sensors have attracted extensive attention in recent years because they have various applications in clinical and environmental analyses and process control as suggested by Badugu et al. [1]. This work reports the new optical chemical sensor for pH determination relying on the immobilization of a combination of two dyes on an optically transpicuous agarose membrane. Kostov and Hashemi reported [2,3] that the immobilization of a combination of two dyes with a variety of p*K*_a_ amounts can expand the desirable dynamic extent of a pH sensor. Here, a mixture of Alizarin and Orange dyes is employed to fabricate a new optode to expand the desirable extent of the pH sensor. The dyes with the intended structure shown in Fig. 1 are covalently immobilized on an epoxy activated support to construct a long life optical sensor for on-line pH monitoring a flow cell.

### Chemicals and reagents

All reagents employed in the current research were of analytical grade. Alizarine, agarose, orange dye, epichlorohydrin, and several other chemicals were purchased from Merck Chemical Company (Darmstadt, Germany). In addition, double-distilled water was utilized, and the intended test solutions were buffered in 0.02 mol L^−1^ sodium dihydrogen phosphate solutions. Further, pH was adjusted by adding 0.5 mol L^−1^ hydrochloric acid or sodium hydroxide solutions.

To determine pH, a pH meter (model 3020, Jenway, USA) with a combined glass electrode was applied after calibration against standard Merck buffers. Moreover, a double-beam spectrophotometer (model 1650PC, Shimadzu, Japan) was employed to run electronic absorption spectra (controlled to ± 0.1 °C). Additionally, a home-made polyacrylamide holder of the flow cell described elsewhere [3] was utilized to hold absorbance measurements and the agarose membrane. In addition, a peristaltic pump (EYLA, Japan) was applied to pump the solutions through the flow cell with a 5 mL min^−1^ flow rate. Faster or slower flow rates were not considered due to an increase in back pressure in the flow system or the instability of the flow cell, respectively. To calculate the response time and calibration curve, absorbance measurements at a constant wavelength were utilized in a similar flow system.

### Preparation and activation of agarose membranes

Overall, 10 mL of the 4 % (w/v) agarose solution was prepared by dissolving agarose powder in boiling water. Then, the hot solution was poured and softly squeezed between two glass plates (20×20 cm) with a distance of 0.2 mm and allowed to be cooled down to room temperature. Next, the obtained membrane, which was transparent and thin, was cut into 3 × 3 cm pieces and underwent cold storage in a 50 % methanol solution. Furthermore, an epichlorohydrin technique was utilized to activate the epoxy [4,5]. Further, about 0.78 mL of 6 % epichlorohydrin and 3.2 mL of 2 mol L^−1^ sodium hydroxide solutions were added to 10 pieces of agarose membranes in a 25 mL beaker, and the obtained solution was diluted to 10 mL by using water. The mentioned mixture was heated to 40 ^º^C in a water-circulating bath and then agitated for 2 h. Following cooling, the membranes activated with the epoxy were completely washed with water on a glass filter and then stored in water at 4 ^º^C. The activated membranes can be stored for a long period prior to dye immobilization.

### Preparation of immobilized optical sensor

In the second technique, to immobilize a combination of Alizarine and Orange dyes on activated agarose membranes, the pieces of the activated membranes were suction dried and then transferred into a beaker consisting of 10 mL solution of a 1:1 proportion of the mentioned dyes with 2.5 × 10^−3^ mol L^−1^ concentrations in 0.02 mol L^−1^ sodium dihydrogen phosphate. Further, the pH rate was adjusted to 11.0, and the mixture was stirred in a 40 ^º^C water bath for 24 h. The obtained optical sensors were perfectly cleaned with water on a glass filter, immersed in water overnight, and finally washed once more with extensive water to replace any non-bond dyes. The membranes were prepared for use and were able to be cut in proper size and mounted in the flow cell. Finally, the cell was applied for determining the absorbance. All the measurements on agarose membranes were conducted in an aqueous medium.

## Analyses

### Preparation of the optical pH sensor

**Fig. 2:**
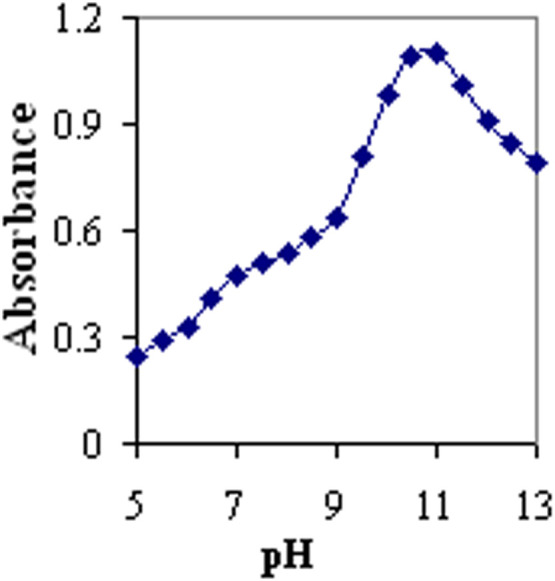
Absorbance of the pH sensor at 490 nm as a function of pH immobilization*. Note*, the concentrations of dyes were 5.0 × 10^−3^ mol L^−1^ with a concentration ratio of 1:1.

The pH impact on the coupling of a 1:1 proportion of Alizarin and Orange dyes is illustrated in Fig. 2. Based on the result, a maximum absorbance was detected at a pH rate of 11.0, which was considered the optimum pH rate.

**Fig. 3:**
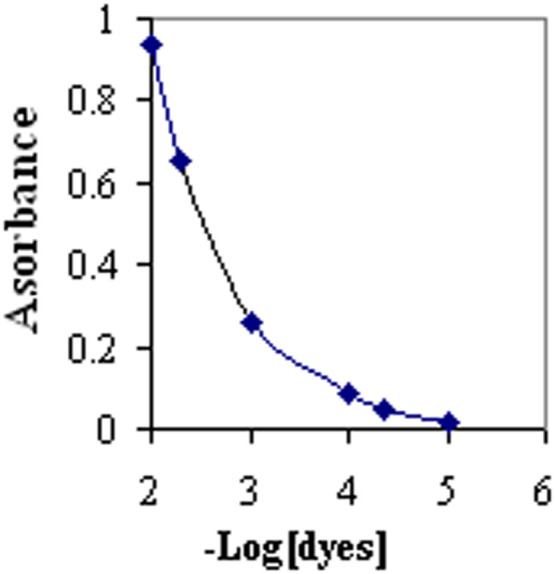
Absorbance of the agarose membrane immobilized at 490 nm as a function related to the concentration of a combination of two indicators of Alizarin and Orange dyes during synthesis.

The concentration impact of Alizarin and Orange dyes with a 1:1 concentration ratio on the sensor absorbance at 490 nm underwent an investigation. Based on the findings (Fig. 3), increasing the dye concentration from 1 × 10^−5^ to 1 × 10^−2^ mol L^−1^ in the solution led to an increase in the absorbance of the agarose (0.016–0.940 absorbance units). Considering that an extremely high absorbance can reduce the optic sensor's transparency while increasing absorbance measurements’ uncertainty, a 5.0 × 10^−3^ mol L^−1^ concentration with a reliable and measurable absorbance rate was utilized in the subsequent experiments. The concentration ratio of both mentioned dyes was another parameter for evaluation. A 1:1 proportion of the intended dyes was chosen as optimum as it yields a sensor with an extensive dynamic range for measuring pH.

### Optical characteristics of the sensors

**Fig. 4:**
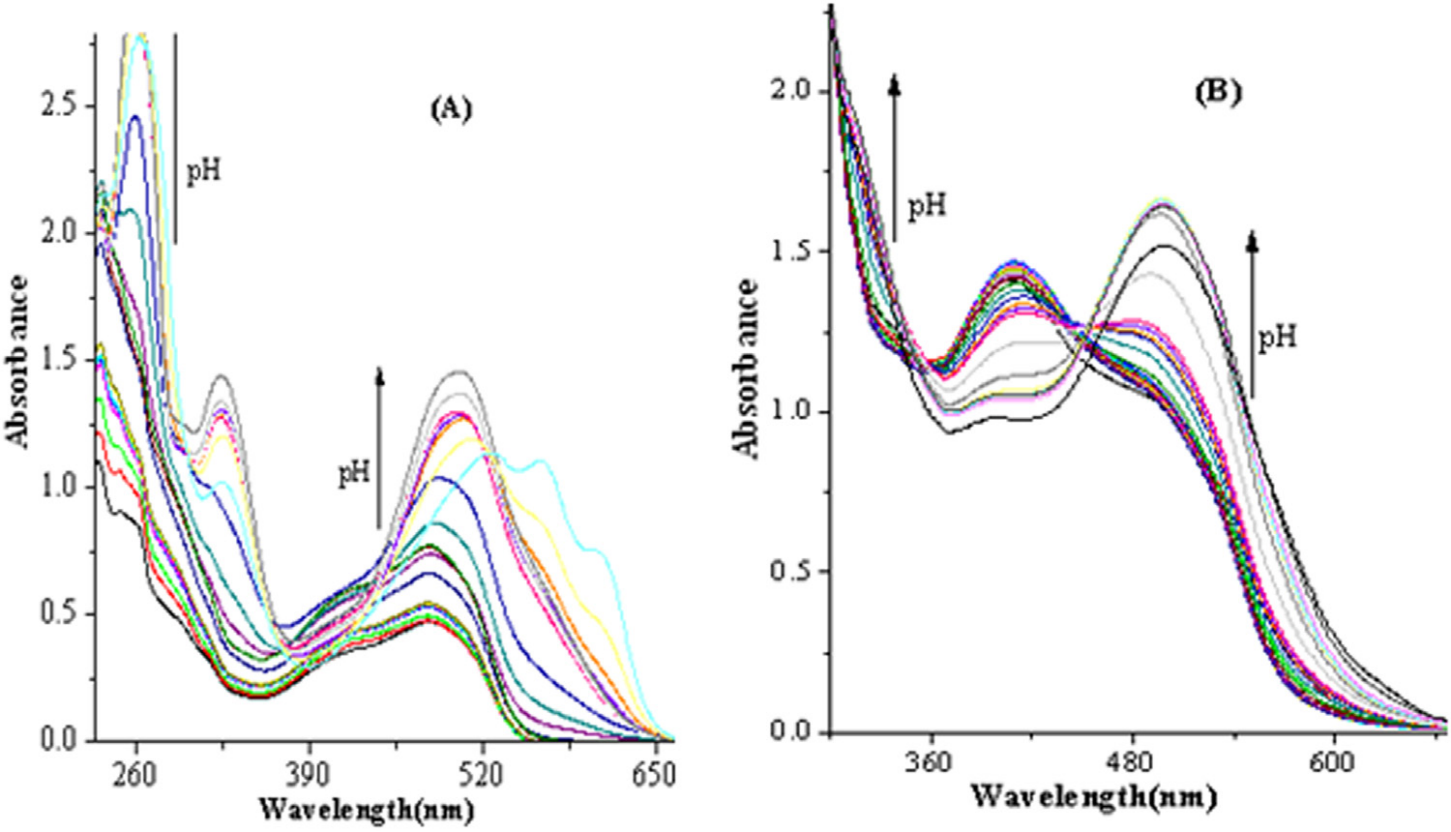
Titration spectra for (A) dissolved and (B) immobilized forms of a combination of the two indicators of Alizarin and Orange dyes at various pH rates. *Note*, the arrows illustrate the direction of absorbance alterations with increasing pH.

Titration spectra for dissolved forms of a 5.0 × 10^−4^ mol L^−1^ combination of two indicators of Alizarin and Orange dyes at various pH rates are depicted in Fig. 4A. Moreover, Fig. 4B displays titration spectra for the immobilized forms of a mixture of two indicators of the mentioned dye upon immobilization on the agarose membrane at different pH rates. As shown, the immobilized and dissolved structures of the dye display noticeable differences in their optical characteristics and acid-based reactivities. The immobilized and dissolved forms of the dye depict a crystal-clear maximum in the detectable region at 500 nm and a slight blue change to 490 nm for the dissolved and immobilized forms, respectively. The relative intensity of the maximum also varies for immobilized and dissolved indicator forms. A less concentrated first maximum for the sensor is probably due to the formation of a chemical bond between the epoxy groups of the agarose support and the functional groups of the two mentioned dyes, leading to a change in its optical characteristics.

### Effect of ionic strength

The impact of ionic strength on absorption was evaluated at of 3 and 6 pH rates. The results revealed that ionic strengths of up to 0.5 mol L^−1^ could not noticeably affect the absorbance of the immobilized Alizarine and Orange dyes at 490 nm, implying that pH can be precisely calculated with this optode in high salt field specimens.

### Calibration curve

**Fig. 5:**
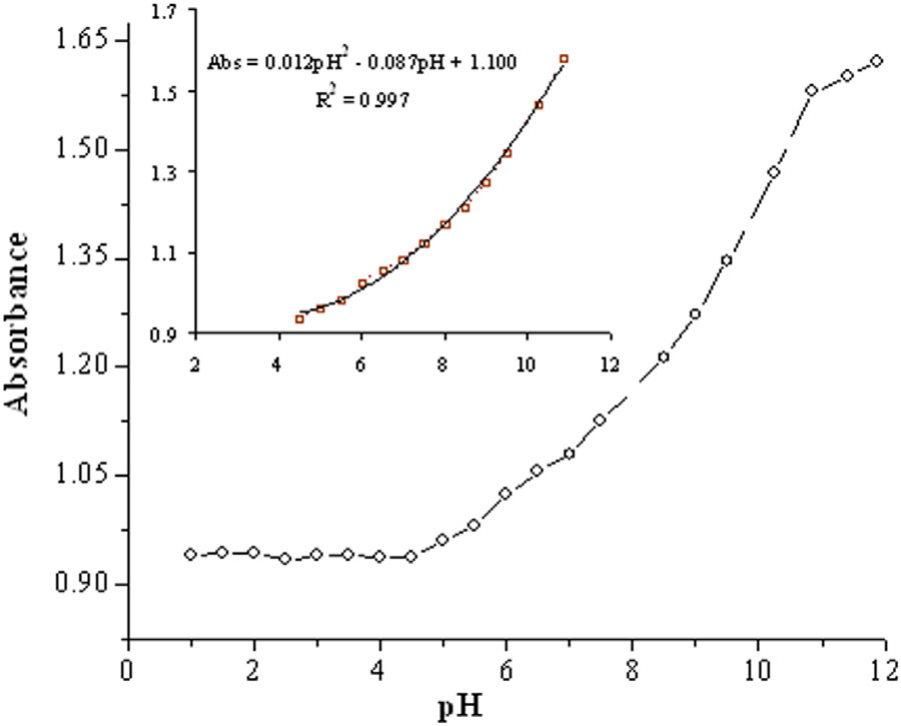
A typical calibration graph obtained from the membrane's absorbance measurements at 490 nm at 1.0–12.0pH rates. *Note*, the inset illustrates the related plot for points that are located on the lineal segment at a pH rate of 4.5–11.

The dynamic working ranges for the suggested pH optode are typically limited to some pH units. According to Safavi and Hashemi [3,6], the relationship between an optode's response and pH is not necessarily linear, and a non-linear multivariate calibration method is required frequently. Fig. 5 depicts a normal calibration graph obtained from the agarose absorbance measurement at 490 nm at a 0.5–12pH rate. The experimental points are perfectly fitted into an equation of Abs. = 0.012pH^2^ −0.087pH+1.100, and an R^2^ value of 0.997 fits the points in the 4.5–11pH range. Accordingly, the fitting is desirable, and the introduced sensor can be simply operated for 4.5–11pH rates. Hashemi and Abolghasemi [3] found that the immobilized Congo Red on the agarose activated with an epoxy could be utilized at a pH rate of 0.5–5.0, along with a polynomial calibration curve. Using a 1:1 proportion of Alizarine and Orange dyes could extend the dynamic range of the pH sensor in this research.

### Reversibility and reproducibility

**Fig. 6:**
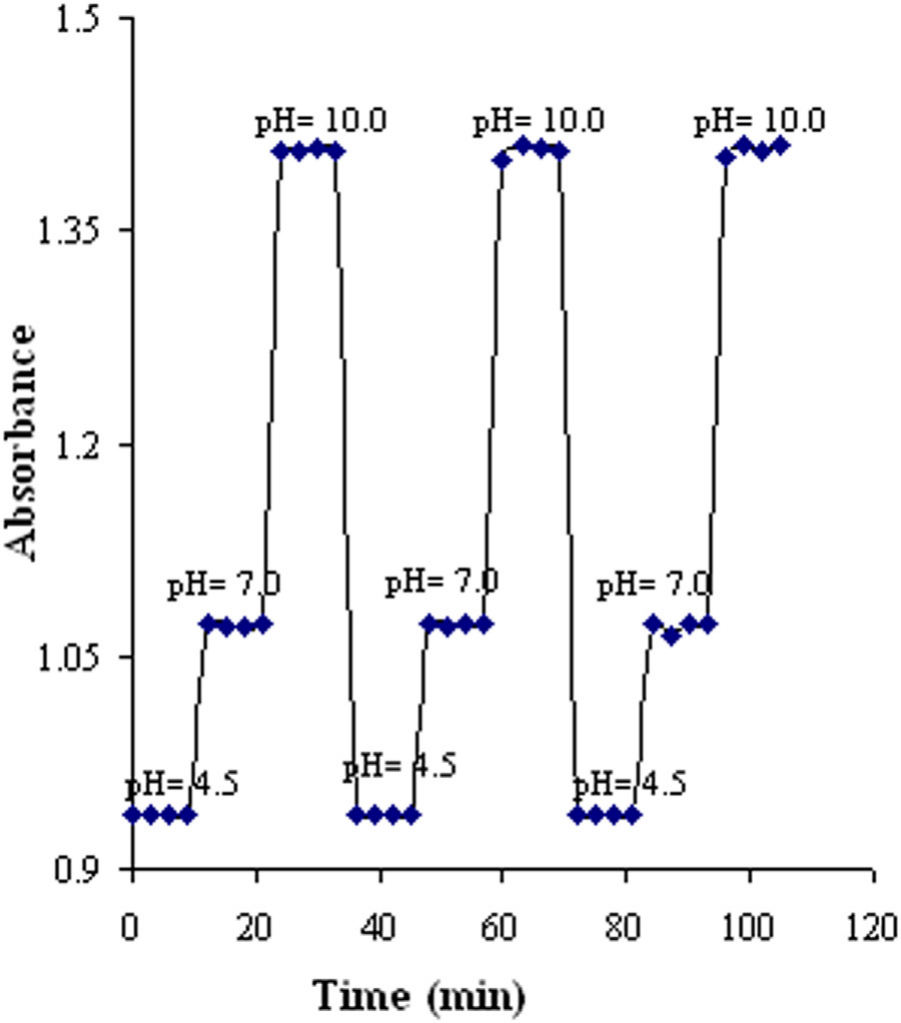
Absorbance variation when the sensor was alternately immersed in solutions with pH rates of 4.5, 7.0, and 10.0.

The essential requirements for an optimal optic sensor include high response sensitivity, fast response time, reasonable reproducibility, good selectivity, and long lifespan. Fig. 6 displays a usual response profile for the pH sensor because of a shift in the pH rate of buffer solutions that pass through the flow cell. The sensor's excellent adjustable response is observable by changing the pH rate between 4.5 and 10.0. The optical signals’ reproducibility was examined by alternately recording the sensor's absorbance change from acidic to basic solutions. The relative standard deviation (SD) was < 0.40 % for a total of six measurements at the tested wavelength.

### Response time

**Fig. 7:**
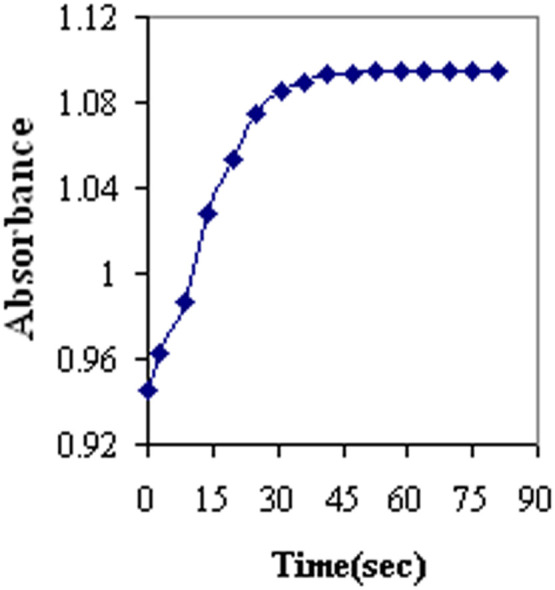
Normal response curve of the sensor at 490 nm as a function of time at the pH rate of 2.5–7.5.

The pH sensor's response time was computed based on absorption profiles at 490 nm. As shown in Fig. 7, the absorbance has reached 95 % of the steady state signal in nearly 25 s. Finally, the signal decreases after equilibrium, and no signal drift can be detected under experimental conditions.

A relative SD < 0.40 % was observed for six replications of absorbance measurements. The impact of dryness on the agarose-based sensor characteristics was evaluated by the dry storage of a membrane during a 3-month period. After 30 min soaking of the membrane in distilled water, the sensor's optical characteristics underwent comparisons with its spectra prior to dryness, and the results represented no meaningful changes in this regard. In addition, no evidence of dye leakage or noticeable change in the pH sensor's absorbance characteristics was found during the 3-month usage.

### Comparison

Table 1 provides the comparison results of the presented sensor with some of the best optic pH sensor reported previously. Considering the benefits of the suggested technique compared to previously introduced methods, it may be employed as an alternative technique for determining pH.

**Table 1:** Comparison between the suggested sensor and some of the best previous optic pH sensors.

**Characterization**	Hashemi et al. [3]	Wang et al. [7]	Safavi et al. [6]	This work
**Sensing Material**	Congo Red	Phenol red	Victoria blue and dipicrylamine	Alizarin and Orange dye
**Type of Sensor**	Agarose membrane	Sol-gel	Triacetyl cellulose membrane	Agarose membrane
**pH range**	0.5–5	6–12	Whole pH range if ANN used	4.5–11
**Response time**	3 min	20 s	56 s	25 s
**Lifetime (month)**	NM^a^	12	NM	more than 3

a not mentioned.

## Conclusions

Overall, a new optic pH sensor was generated by activating an agarose membrane by an epoxy and chemically immobilizing a combination of two indicators of Alizarin and Orange dyes. The new sensor could be applied over a pH range of 4.5–11 and quickly responded to pH alterations in 25 s. The sensor's response was adjustable and replicable. Based on the findings, ionic strengths of up to 0.5 mol L^−1^ failed to significantly affect the response signal. Eventually, the chemical immobilization of two indicators on agarose membranes activated by an epoxy could lead to a sensitive optic pH sensor for a wide range of pH with desirable reproducibility, a short-term response time, and long-term steadiness.

## Ethics statements

The present article contains no studies with human participants or animal experiments by any of the authors.

## CRediT authorship contribution statement

**Kamal Alizadeh:** Visualization, Data curation, Formal analysis, Writing – review & editing. **Behrooz Rezaei:** Data curation, Formal analysis, Writing – review & editing. **Hadi Nemati:** Data curation, Formal analysis.

## Declaration of Competing Interest

The authors declare that they have no known competing financial interests or personal relationships that could have appeared to influence the work reported in this paper.

## Data Availability

The data that has been used is confidential.
